# Students’ Perception of Teachers’ Reference Norm Orientation and Cheating in the Classroom

**DOI:** 10.3389/fpsyg.2021.614199

**Published:** 2021-02-04

**Authors:** Tamara Marksteiner, Anna K. Nishen, Oliver Dickhäuser

**Affiliations:** ^1^Department of Psychology, School of Social Sciences, University of Mannheim, Mannheim, Germany; ^2^Department of Education and Psychology, Freie Universität Berlin, Berlin, Germany

**Keywords:** students’ cheating, deception in school, individual reference norm orientation, mastery goal structure, doubly manifest multi-level analysis

## Abstract

Students’ cheating is a serious problem: It undermines the chance to adequately promote, support, and evaluate them. To explain cheating behavior, research seldom focuses on perceived teachers’ characteristics. Thus, we investigate the relationship between students’ cheating behavior and an important teacher characteristic, individual reference norm orientation (IRNO; i.e., the tendency to evaluate students based on their performance development over time). We examined cheating on written exams, on homework, and in oral exams among *N* = 601 students (64.2% girls; *M*_age_ = 16.07 years) in *N* = 31 language classes. Results from doubly manifest multi-level analyses showed that, on the classroom level, cheating on written exams and on homework occurred less frequently the more the classroom of students perceived their teachers as having an IRNO. We found no further evidence for other cheating factors or student characteristics. This supports the idea that teacher characteristics are associated with some forms of students’ cheating behavior.

## Introduction

Data from 35 nations worldwide indicate that, on average, school principals consider cheating a serious problem (Miller et al., 2015). Cheating – deceitful or fraudulent behavior that unfairly benefits the cheater (Evans et al., 1993) – not only leads to unfair grading of students’ performance, but also to difficulties among teachers to adequately respond to students’ performance and knowledge level. Cheating behavior may include a wide variety of behaviors such as the use of crib notes and plagiarism (Evans et al., 1993; Anderman and Danner, 2008). In order to reduce cheating behavior, we need to learn why some students cheat in some classes and others do not. Previous research on cheating has focused on the impact of *student characteristics* like students’ gender (e.g., Whitley et al., 1999) or their prior performance (e.g., Rost and Sparfeldt, 2003; for an overview on cheating-related factors, see Sparfeldt, 2018). For example, results of a meta-analysis reveal that men reported having cheated to a slightly greater degree than women (Whitley et al., 1999). Further, research has investigated *context characteristics* – factors that could potentially affect all students in the classroom – of the immediate testing situation like handing out parallel versions of a test or strict supervision during the test (e.g., Cizek, 1999, 2003). However, students may form cheating decisions before the testing situations because of factors that are more distant to the actual testing situation and determine the learning environment in a certain way. One can assume that especially *teacher characteristics* which are closely related to the evaluation of students’ achievement, may influence their cheating. Teachers differ in their pursuit of an *individual reference norm*, the tendency to evaluate students based on the development of their (individual) performance over time (Lüdtke et al., 2005). This tendency may be especially important in determining cheating behavior as it directly relates to teachers’ evaluation of students’ performance. Evaluating students’ development over time is one dimension of a *mastery goal structure* (i.e., classroom culture that emphasizes learning and mastery over good grades and competition; Ames, 1992; Meece et al., 2006). In an environment where teachers create a mastery goal structure and pursue an individual reference norm, cheating should be discouraged and occur less often. One reason might be that students’ fear of failure may be reduced (see also Rheinberg, 1983; Mih and Mih, 2016). Anderman et al. (1998) investigated the relation between reported cheating behavior and students’ perception of the goal structures promoted in their school and classroom. Their results indicate that students who perceived an emphasis on performance rather than on mastery and improvement reported cheating. However, at a single school, Anderman et al. (1998) assessed students’ perception of their *school’s* as well as their *science classroom’s* focus on performance or mastery. They analyzed the relation between perceived school or classroom goal structure and cheating behavior on the *individual* student level – not on the *context* or classroom level. Studies including students’ shared perception of the teacher – a context variable – found that mastery goal structures were related to lower levels of cheating (Tas and Tekkaya, 2010). In addition to a lack of studies investigating context effects, research linking goal structures to cheating has not yet taken a more detailed look at which dimensions of a goal structure may drive the effect. This lack of research diminishes our ability to identify powerful causes of cheating as well as factors that may deter students from seeing cheating as a valid option or necessity from the very beginning.

The present study adds to the existing research in multiple ways. First, it aims to establish the relation between one dimension of teachers’ perceived mastery goal orientation, teachers’ perceived *individual reference norm orientation* (IRNO), and cheating. Secondly, we include the students’ shared perception of their teachers’ IRNO (i.e., class-averaged IRNO) in addition to the relation between the students’ personal perception of their teachers’ IRNO (i.e., perceived IRNO) and their reported cheating behavior. To our knowledge, the present study is the first to treat IRNO on the individual student as well as the classroom level as a predictor in a multi-level design and to relate it to students’ reported cheating behavior on both levels.

### Cheating Behavior and Individual Reference Norm Orientation

The question of why students cheat has intrigued many researchers who have investigated perceptions and characteristics of students themselves as well as their environment. In the past, researchers identified a small number of context factors. For example, higher levels of peer cheating and lower peer disapproval of cheating relate to a higher occurrence of cheating (McCabe and Trevino, 1997). Further, school honor codes as well as a moral climate, among others indicated by instructions not to cheat, have been linked to reduced cheating (Bushway and Nash, 1977; Whitley, 1998; Bing et al., 2012). Cheating also seems to be linked to contextual social structures (Paccagnella and Sestito, 2014): Researchers identified cheating as a sign for low trust in authorities and a general disrespect for social norms. While these findings speak to the importance of context factors for cheating, research has seldom investigated how teacher characteristics (e.g., measured by shared student perception) are related to cheating behavior as of yet.

A teacher characteristic that is closely tied to the exam situation in which students cheat is the *reference norm orientation*, (i.e., the standard to which a teacher compares a student’s performance; Rheinberg, 1983). Teachers may make comparisons with an absolute standard (criterial reference norm), with the results of other students (social reference norm), or with the prior performance of the individual student (individual reference norm; Rheinberg, 1983). The preference for an individual reference norm has been linked to positive effects for students: For example, research found that IRNO buffers the decline in students’ self-concept (e.g., Dickhäuser et al., 2017) and implicit theory of math ability during adolescence (e.g., Dweck, 1986; see also Lüdtke and Köller, 2002), while a social reference norm orientation was accompanied by an accelerated decline. Retelsdorf and Günther (2011) reported that teachers’ IRNO impacted their instructional practices, and could thus influence how much students can learn in class. Moreover, Rheinberg (1983) reports that students with low abilities seem to especially benefit from their teacher’s IRNO: In a longitudinal study with 193 students, Rheinberg (1983) found that 5th graders reported less fear of failure at the end of the school year if their teacher preferred an IRNO at the beginning of it. This effect was more pronounced for those with the lowest academic achievement. Further, in a cross-sectional study of high-school students, Mih and Mih (2016) found that students who reported higher fear of failure also reported cheating more in school (e.g., plagiarism and cheating on tests). This relationship was found to be mediated by disaffection (behaviors indicating disengagement) and procrastination, though this should be replicated in a longitudinal design to establish temporal precedence.

Students who fear failure perceive threat and feel anxious in situations that involve the possibility of failing (Mih and Mih, 2016). This fear could motivate them to either avoid the situation or to reduce the likelihood of failing – for example, by cheating. Thus, the teacher’s use of evaluation practices that emphasize ‘individual progress, improvement, and mastery (Meece et al., 2006, p. 493) may be a meaningful factor in students’ cheating behavior.

If students feel that they are judged by their individual improvement rather than a criterion or the performance of others over which they have no control, they might be less afraid of failing (Rheinberg, 1983). Moreover, failure might become less common because individual improvement is within students’ reach, unlike passing other students in class to be at the top.

Additionally, students’ perceived need to cheat in order to gain an advantage over others or meet a specific target may be lower if their teacher has or is perceived to have an IRNO. Previous research indicates that teachers with a high IRNO respond to students’ performance based on their specific learning curves rather than based on a comparison to other students or a fixed criterion (Meece et al., 2006). Students may perceive teachers with a pronounced IRNO as noticing every single student’s improvement or decline in understanding and performance. As a result, students may start using an IRNO to assess their own performance. In this case, cheating would be counterproductive because it does not allow them to correctly chart their own progress.

To our knowledge, no research has yet directly investigated the relationship between a teacher’s IRNO and their students’ cheating behavior. However, the general mastery goal structure, of which the IRNO is an important dimension (Meece et al., 2006), has been investigated in relation to cheating. Therefore, we discuss the more general connection between mastery goal structures and cheating behavior.

### Goal Structures

As the more comprehensive construct including multiple aspects of teachers’ behaviors, the (perceived) *goal structure*, and its relationship to cheating has received more attention (e.g., Anderman, 2007; Anderman and Koenka, 2017; see also Daumiller and Janke, 2020, on the effect of performance goals on academic cheating), both as student-level and classroom-level predictors. Goal structures represent the emphasis that one puts on goals in a specific context (e.g., a class). Recent research distinguishes between *mastery* and *performance goal structures* (Ames, 1992; Meece et al., 2006; Anderman, 2007). In a class with a *mastery goal structure*, the teacher emphasizes skill development, understanding of the material, and personal progress. In contrast, if teachers adopt a *performance goal structure*, they stress the importance of grades, competition, and social comparison.

Research focusing on students’ personal perceptions of goal structures indicates that mastery goal structures are beneficial for students compared to performance goal structures (Meece et al., 2006). Existing research has linked perceived mastery goal structures to less use of avoidance strategies (Turner et al., 2002), higher mastery goal orientations (Wolters, 2004; Bong, 2008) as well as to the use of more effective learning strategies (Ames and Archer, 1988). Additionally, mastery goal structures are generally associated with less cheating (Anderman, 2007; Anderman and Danner, 2008; see also Anderman and Koenka, 2017). In their vignette study, Murdock et al. (2004) found that students rated cheating as less justifiable and less likely in classrooms described as having mastery goal structures than in classrooms with performance goal structures. In a study of students transitioning from middle school to high school, Anderman and Midgley (2004) found that students who moved from a classroom with low perceived mastery goal structures to a classroom with higher mastery goal structures reported cheating less after the transition. Bong (2008) found that the more students perceived their math classroom to have a mastery goal orientation, the greater their self-efficacy was, and the less they cheated in their math class.

To a lesser extent, students’ perceptions of teachers’ mastery goal structures have been treated as genuine context factors rather than individual perceptions. This is to say that researchers averaged students’ perceptions to form an *aggregate measure* reflecting the shared perception of teacher characteristics and the general climate prevailing in a classroom. The procedure to aggregate students’ personal perceptions to approximate a climate is common among research on context effects (e.g., Lüdtke et al., 2005; Tas and Tekkaya, 2010). These aggregate measures permit the study of students’ perceptions rather than the potentially biased self-ratings of teachers, while at the same time correcting for the potential individual biases in each student’s perception.

In a large correlational study with 1,950 7th grade students, Tas and Tekkaya (2010) found that higher aggregated perceived mastery goal structures were associated with less cheating on the classroom level. In their multi-level analyses, they tested student level variables (e.g., goal orientations, perceived goal structure of the classroom, self-efficacy) as predictors of students’ cheating. Additionally, they predicted differences between classrooms using the aggregated classroom mastery goal structure. This effect was significant, demonstrating that classrooms that were collectively perceived as promoting a mastery goal structure also had lower frequency of cheating. Notably, the aggregated measure accounted for 23.4% of the between-class variance. Interestingly, students’ individual perceptions of the classroom mastery goal structure were unrelated to their own cheating (student level). Students’ individual cheating was instead predicted by their self-efficacy, self-handicapping strategies, and goal orientations.

The relation between mastery goal structures and cheating has usually been explained in terms of reduced motivation to cheat. Anderman and Danner (2008, see also Anderman and Koenka, 2017) suggest that cheating is a strategy for goal attainment – a strategy that is not useful when developing new skills is the goal. Not only will cheating hinder the achievement of the student’s goal, but it will also mask important indicators of their progression toward the goal of development. Overall, the evidence suggests that mastery goal structures can powerfully influence students’ cheating behavior. While this informs our general notion of their importance, it is still unclear which aspects of mastery goal structures may contribute to the effect. Since reference norm orientations are separable from more comprehensive goal structures, they need to be investigated as predictors of cheating in their own right.

### The Present Study

Since the 1990s, research has investigated cheating behavior more thoroughly. Researchers used Achievement Goal Theory (e.g., Anderman, 2007) to evaluate factors influencing students’ cheating behaviors on the student level. To date, research on the impact of goal structures has found that a mastery approach to teaching diminishes individual students’ cheating behavior (Anderman and Midgley, 2004; Murdock et al., 2004; Bong, 2008). While an IRNO has not yet been directly linked to cheating, it may reduce fear of failure, which in turn may reduce the perceived necessity of cheating (Rheinberg, 1983). In sum, an increased mastery goal structure and with it an increased IRNO can presumably reduce the motivation to cheat because it hinders students’ goal attainment and diminishes the perceived pressure to gain an advantage over others.

The research presented in this article aims to add to the existing literature by focusing on one aspect of a mastery goal structure (i.e., teachers’ IRNO). Teachers’ IRNO might be a particularly powerful predictor of students’ reported cheating behavior since it is a central aspect of the evaluation situation in which students may decide to cheat and may reduce one central motivator to cheat: fear of failure. Therefore, we investigate the relation of students’ personal perception of their teacher’s IRNO with their reported cheating behavior. Additionally, we aim to investigate another predictor of cheating (i.e., perceived IRNO on the classroom level), alongside students’ personal perception of their teacher’s IRNO. Although social norms about cheating and peers’ cheating behavior influence students’ cheating (McCabe and Trevino, 1997), research has not yet investigated aggregated perceptions of teachers’ IRNO. We hypothesize that the more students in a class collectively perceive their teacher to have an IRNO, the less they report cheating. We also assume a negative relationship between the perceived IRNO and reported cheating on the student level. Because an IRNO should be endorsed to the same degree in different testing situations by the teacher, and therefore could reduce fear of failure independent of it, we expected all three aspects of cheating that we assessed (i.e., written exams, homework, and oral exams) to be impacted equally.

## Materials and Methods

### Participants

In total, *N*_classes_ = 31 classes and all of their respective students (*N*_students_ = 601; 64.2% girls) answered the questionnaires. Average class size was 19 students (*SD* = 7.15; *range* = 6–35). In Germany, primary and lower secondary education lasts for 9 or 10 years in total. During primary education, schools are not tracked, whereas lower secondary education comprises schools that are tracked according to students’ ability level. Even though there is some degree of variation between German federal states concerning the number (mostly two or three) or types of tracks, in all federal states there exists a highest-level track, called Gymnasium. The lower-level tracks put a stronger focus on vocational education and students graduating from these schools are more likely to enter apprenticeships afterward. Döbert (2015) gives a more detailed overview of the German School system. Academic track secondary schools, in particular, offer students a high variety of language classes, while most Gymnasiums offer Latin, English, and French. However, at some schools, students can also choose other language classes (e.g., Spanish or Japanese), or have certain classes taught in a foreign language. In the present study, schools were mostly academic track secondary schools (74.2%), but also vocational schools (22.6%), and one integrative secondary school (3.2%). The majority of foreign language classes were English (41.9%), Spanish (25.8%), and French classes (19.4%). The rest of the classes were Latin (6.5%), Japanese (3.2%), and Geography in French classes (3.2%). On average, the students were *M*_age_ = 16.1 years old (*SD* = 1.91; *range* = 11–22) and were in 10th grade (*SD* = 1.6; *range* = 7–13). In total, 15.1% of students reported that German was not their first language.

### General Procedure

In two federal states of Germany, we asked language teachers and the students of their language class to participate. We collected the data during the middle of the academic year. All students’ parents expressed informed consent before participation. First, students created a personal code (i.e., first letter of their first name, second letter of their last name, and their birthday). Then, students indicated their type of school, [e.g., comprehensive secondary school (Gymnasium), vocational school], the federal state of their school, their class level, their age (in years) and gender (0 = male, 1 = female), and for how long they had had their current language teacher. Next, they answered questions about their cheating behavior and their perception of their teachers’ IRNO. Further, they answered questions on how often they had lied to their language teacher on different occasions, as well as how caring and trustful their teacher is. Teachers indicated their type of school, the federal state of their school, the amount of teachers at their school, and what kind of classes at which class level they teach. Then, they reported demographic characteristics such as gender, age, years of teaching experience, and for how long they had taught their current language class. Next, teachers answered questions regarding their trust and suspicion toward students as well as their caring behavior, and IRNO. As an incentive, students were able to participate in a lottery where one out of every 30 students won an MP3-Player. Participation in the study took approximately 20 min.

Either the investigator or the participating teacher administered the questionnaires to their students. Participants were assured that they could quit the questionnaire at any time and that all of their responses would remain confidential. After answering all questions, students sealed their answers in an envelope. Envelopes were gathered and, afterward, handed over to the investigator or their teacher. The study was conducted in full accordance with the Ethical Guidelines of the German Association of Psychologists (DGPs) and the American Psychological Association (APA). At the time the data was acquired, it was also not customary at the respective university, nor at most other German universities, to seek ethics approval for survey studies on teachers’ goal orientation and reported cheating behavior. The questionnaires are anonymous, thus, no identifying information was conducted. Further, we had no reasons to assume that our survey would induce persisting negative states (e.g., clinical depression) in the participants or have other forms of negative consequences for participants.

### Measures

#### Cheating Behavior

Students filled out an adapted 23-item version of a cheating scale for students developed by Rost and Wild (1990) using a scale ranging from 1 to 5 (1 = *never applies*, 2 = *almost never applies*, 3 = *sometimes applies*, 4 = *almost always applies*, and 5 = *always applies*). The scale assessed cheating in three different testing situations: *Cheating on written exams* was assessed by seven items (e.g., “When I am writing a quiz or exam in my language teacher’s class, I write a crib note”; Cronbach’s α = 0.74). *Cheating on homework* was assessed by seven items (e.g., “When my language teacher controls my homework, I forget my homework on purpose”; Cronbach’s α = 0.75). *Cheating in an oral exam* was assessed by three items (e.g., “When I have an oral exam in my language teacher’s class, I insist on my wrong answer and claim that it is what I had heard in class”; Cronbach’s α = 0.76). Due to the broad definition of cheating by Rost and Wild (1990), items not explicitly reflecting cheating (intentions) or deceitful behavior (e.g., “When I am writing a quiz or exam in my language teacher’s class, I sit down next to a good student”) had to be excluded from the analyses after thorough discussion.

#### Perceived IRNO

To assess teachers’ IRNO, we used the German validated scale by Schwarzer et al. (1982). The scale consists of four items and ranges from 1 = *does not apply* to 4 = *applies exactly* (e.g., “My language teacher always notices immediately when my performance gets better or worse” or “Our language teacher praises even the worst students when he realizes that they have improved”). Higher values indicate a higher perceived IRNO. In our sample, the internal consistency of this scale was 0.65.

#### Class-Averaged IRNO

To measure the general perception of the teacher’s IRNO in a given classroom, we averaged the scores of the students of the scale by Schwarzer et al. (1982). As described earlier, researchers often follow this aggregation procedure to approximate a climate or context when the personal perceptions – of teachers or students – may be more biased.

### Analytical Procedure

First, to establish the extent to which cheating and IRNO depended on the classroom, we calculated the intraclass correlation ICC(1) for each of the factors of cheating as well as for perceived IRNO using the statistical software MPlus 7.2 (Muthén and Muthén, 2017). To establish the reliability of the class-averaged student ratings, we calculated the intraclass correlation ICC(2) (see Lüdtke et al., 2006, for more information on the intraclass correlation).

To test the hypothesis and to replicate previous findings, we specified a doubly manifest multi-level model in MPlus (Marsh et al., 2009) including both the student level and the aggregated classroom level using the ANALYSIS: TYPE = TWOLEVEL command and the estimator MLR (see Muthén and Muthén, 2017). According to the *N*:*q* rule and a recommended sample-size-to-parameters ratio of 20:1 (see Kline, 2016, p. 16, for more information), our sample size is sufficient for a doubly manifest (but not a latent) multi-level model. To correct for measurement errors in the single-indicator variable, we followed the approach described by Wang and Wang (2019, see also Sagan and Pawelek, 2014). Previous research indicates that male students report more cheating behavior than female students (e.g., Whitley, 1998), while other studies did not find relevant gender differences (e.g., Rost and Sparfeldt, 2003). Therefore, we included gender as a covariate in our model.

## Results

### Descriptive Statistics

The mean reported cheating ranged between *M* = 1.36 (*SD* = 0.43) and *M* = 1.80 (*SD* = 0.77) on the three scales (see Table 1), indicating rather low absolute levels of cheating. Table 1 displays means and SDs for the three cheating scales and perceived IRNO. Table 2 displays correlations between these variables on the student and class level. The three cheating factors correlated positively with one another on both levels (see Table 2). Cheating on written exams and cheating on homework were significantly related to perceived IRNO on both levels. All significant correlations were notably higher on the classroom level.

**Table 1:** Means, SD, ICC(1) and ICC(2) of cheating factors and perceived individual reference norm orientation (IRNO).

	Student level		Classroom level		ICC(1)	ICC(2)
	*M*	*SD*	*M*	*SD*		
Cheating on written exams	1.59	0.52	1.58	0.21	0.12	0.73
Cheating on homework	1.36	0.43	1.36	0.13	0.05	0.51
Cheating in oral exams	1.80	0.77	1.79	0.28	0.12	0.73
Perceived IRNO	2.79	0.28	2.80	0.27	0.20	0.83

IRNO, Individual reference norm orientation. ICC(1) represents the reliability of an individual student assessment and ICC(2) represents the reliability of the class-averaged student ratings (Lüdtke et al., 2006).

**Table 2:** Correlations between cheating factors and IRNO on student level and classroom level.

	IRNO	Cheating on written exams	Cheating on homework	Cheating in oral exams
IRNO		−0.443^*^	−0.399^*^	−0.061
Cheating on written exams	−0.205^***^		0.664^***^	0.410^*^
Cheating on homework	−0.127^**^	0.567^***^		0.562^**^
Cheating in oral exams	−0.066	0.388^***^	0.445^***^	

Values beneath the diagonal represent correlations on the student level. Values above the diagonal represent correlations on the classroom level.

* *p* < 0.05;

** *p* < 0.01;

*** *p* < 0.001.

### Intraclass Correlation and Multi-Level Reliability

In the empty model, all cheating factors had an ICC(1) larger than 0.01 (range = 0.05–0.12; see Table 1), suggesting a dependency of students’ reported cheating behavior on their classroom, and an ICC(2) larger than 0.50 (range = 0.51–0.73), suggesting a sufficient to good reliability of the class-averaged student ratings. In addition, perceived IRNO showed a substantial variation between classes, ICC(1) = 0.20. This indicates that a significant part of their variance can be explained by differences between classrooms. Further, perceived IRNO had an ICC(2) of 0.83, indicating a good reliability on the class level. Therefore, in a next step, we used a doubly manifest multi-level model to predict the variation of cheating at the student as well as at the classroom level.

Further, we computed w and Maximal Reliability (H) for IRNO on the individual as well as on the class level following Geldhof et al. (2014, see also https://www.franciscowilhelm.com/post/how-to-compute-multi-level-reliability-indices-in-r-and-mplus/). On the individual level, the results indicate a low to acceptable reliability (w_within_ = 0.621, *p* < 0.001; H_within_ = 0.664, *p* < 0.001). On the class level, the results indicate a good to very good reliability (w_between_ = 0.895, *p* < 0.001; H_between_ = 0.924, *p* < 0.001).

### Doubly Manifest Model

**Figure 1:**
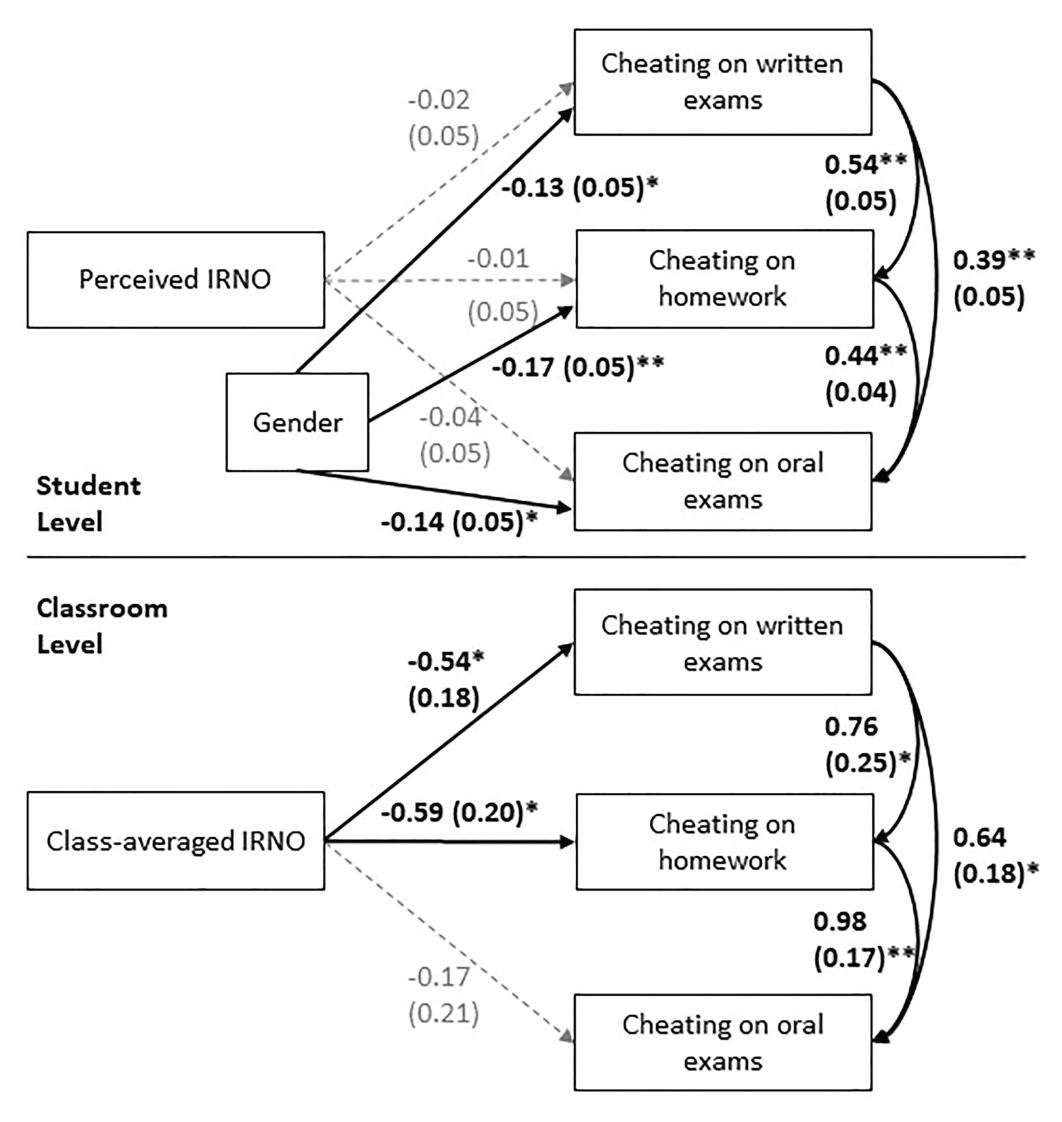
Results of the doubly manifest multilevel model. Standardized regression coefficients are displayed. Values in brackets represent standard errors of the coefficients. Significant pathways (*p* < 0.05) are presented in bold. ^**^p < 0.001; ^*^p < 0.05.

The model, which also included gender as a covariate, showed very good model fit, *RMSEA* = 0.037; *CFI* = 0.998; *TLI* = 0.974; *SRMR*_within_ = 0.013, *SRMR*_between_ = 0.005; *AIC* = 3753.27; and adjusted *BIC* = 3868.49. Typically, values over 0.95 (TLI and CFI) and values below 0.05 (RMSEA and SRMR) are considered to indicate good fit (commonly based on Hu and Bentler, 1999; but see Sivo et al., 2006, for a detailed discussion of cut-off values under varying conditions). To be able to compare the goodness of fit of the null model with the full model and given the nested data structure, we computed the likelihood ratio test (LRT) following the instructions on the Mplus website (see http://www.statmodel.com/chidiff.shtml for additional information). The results of the LRT indicate a statistically significant difference between the nested (null) model and the full model, *𝜒*^2^(9) = 1,959.92, *p* < 0.001, and better fit of the full model (compared to the null model) to the data. Figure 1 displays the standardized path coefficients and the significant as well as non-significant pathways.

#### Teachers’ Perceived IRNO and Reported Cheating

The multi-level model specified a relationship between perceived IRNO and reported cheating in all three situations (written exam, homework, and oral exam). The results indicate that perceived IRNO did not relate to cheating in any situation on the student level (Figure 1), all *p*s > 0.50. Participant’s gender was significantly related to all three cheating factors, all *p*s < 0.05, indicating that male students reported more often having cheated.

#### Class-Averaged Teachers’ IRNO and Cheating

On the classroom level, we assumed that perceived IRNO on the classroom level inversely relates to reported cheating in each situation (Figure 1). A look at the pathway coefficients revealed that the higher a class perceived their teachers’ IRNO to be, the less they reported cheating on written exams (*β* = −0.55, *p* = 0.011) and on homework (*β* = −0.59, *p* = 0.003). Class-averaged IRNO was not associated with reported cheating in oral exams (*β* = −0.17, *p* = 0.40) in a statistically significant way.^1^

## Discussion

The aim of the present study was to investigate the relationship between students’ reported cheating behavior and their perceptions of their teachers’ IRNO. We hypothesized that a higher perceived IRNO would be associated with less reported cheating on the classroom and student levels.

As expected, we found a negative relationship between IRNO and reported cheating on written exams and on homework on the classroom level. The higher students collectively perceived their teachers’ IRNO to be, the less they reported cheating on written exams and on homework. This result mirrors the findings of prior research on the effect of mastery goal structures more generally (e.g., Tas and Tekkaya, 2010). It provides a first indication that the evaluation dimension of mastery goal structures is central to the relationship of general mastery goal structures with cheating. Fostering students’ collective sense that their personal development over time is important to the teacher may send the message that cheating is not effective to achieve one’s goals. Future research should determine the exact process by which the more distal factor of collectively perceived IRNO is associated with students’ decision to cheat (e.g., through fear of failure).

Notably, the collectively perceived IRNO was not related to reported cheating in only one situation: in oral exams, students did not report to cheat less when they collectively perceived their teachers’ IRNO to be higher. This might be due to the nature of written exams and homework: Cheating on written exams and, especially on homework, should be easier than cheating during oral exams – not least because the latter is an individual testing situation in which answers must be given immediately. Thus, cheating on oral exams should be harder and more easily detected than cheating on written exams and on homework. Therefore, other factors may more strongly relate to students lying to their teacher in order to get a better grade. Importantly, the items of the subscales for cheating on oral exams reflects making fraudulent excuses (e.g., “When I have an oral exam in my language teacher’s class, I act as if I knew the answer a second ago,” but note that this could also apply to cheating on homework). Fraudulent excuses represent a facet of academic dishonest behavior that is often distinguished from other facets, namely cheating on exams and plagiarism (Roig and Caso, 2005; Hensley et al., 2013). In their study, Roig and Caso (2005) found that fraudulent excuses are prevalent (72% of the sample report at least one incident), and mainly motivated by efforts to win time and move a deadline. Thus, using fraudulent excuses may not necessarily be motivated by an effort to illegitimately receive a better grade, but rather to feel better prepared for an examination and put one’s best foot forward (and receive a better grade as a result). Therefore, other dimensions of goal structures may be more helpful to understand cheating in oral exams. Specifically, Ames (1992) denotes a *timing dimension*, which indicates to what degree students have the opportunity to plan their assignments and have adequate time to work on them. Overall, differences both in what motivates the dishonest behavior and what it requires (e.g., lying directly to a teacher) may explain the differential effects of IRNO for the different types of cheating.

On the student level, the analyses did not reveal any significant relationships between perceived IRNO and reported cheating in any of the three cheating situations. While these results regarding the evaluation dimension of goal structures do not stand in direct contrast to prior findings on overall mastery goal structures, they are somewhat surprising given the reliable findings connecting mastery goal structures and cheating. Interpreting this result in relation to existing research, a dimension of mastery goal structures other than the evaluation dimension might be responsible for their positive relation with cheating on the student level. For example, incentives and rewards given for accomplishments might be more relevant to individual students (*recognition dimension*; Meece et al., 2006). In their multi-level analysis, Tas and Tekkaya (2010) found that though perceived mastery goal structures were related to less cheating on the classroom level, students’ perception of the classroom goal structure was not related to their own cheating. Rather, students’ personal goal orientations predicted their own cheating behavior. Our results replicate this finding that the perception of classroom goal structure is relevant only to explain differences in cheating levels across classrooms rather than across individuals.

Although our results are compatible with existing research, the question remains why a collective perception of IRNO was associated with the collective level of cheating in a written exam in the class, but the individual perception of IRNO was not associated with individuals’ cheating in written exams. Potentially, the decision to cheat on an individual level is associated with more qualifications of the effect of IRNO, rendering it impossible to detect its effect without accounting for moderators. For example, situational and stable factors which differ from student to student (e.g., self-efficacy, opportunity to cheat, social norms in students’ cliques) may impact individuals’ decision to cheat more so than a – relatively stable – perception of one’s teacher. Since these factors vary across students, they may be similar across classrooms and allow the effect of IRNO on cheating on written exams and on homework to be observed.

### Limitations and Further Research

It is important to mention some limitations of the present study. Importantly, the number of variables assessed in the study was restricted and, as a result, we were unable to compare the relation of the IRNO to that of social and criterion reference norm orientations. An inclusion of these constructs would have allowed us to make more detailed observations regarding the relation of cheating with reference norm orientations. However, insight into the role of the IRNO has allowed us to suggest practical implications for teachers below.

Moreover, we were not able to assess the relationship of teachers’ IRNO in relation to other known factors with cheating behavior of students. This mono-causal analysis – though differentiating the predictor on two different levels – does reduce the generalizability of our study, as we cannot determine the conditionality of our results. Notably, research findings often connect academic achievement to cheating. Specifically, students low in academic achievement may have a higher motivation to cheat – as they are less able to meet the test criteria through honest means. High-achieving students may also cheat when encountering challenging tasks (Anderman and Danner, 2008). Unfortunately, we did not assess achievement as a control variable in this study. Lower-achieving students may be more strongly impacted by their own and the shared perception of teachers’ IRNO since they may be especially afraid of failure. Future studies could explore whether academic achievement may additionally affect the relationship between the teacher’s perceived IRNO and cheating behavior as a moderating variable.

Importantly, the reliability of the scales in the sample limited our ability to detect associations between the constructs. Because low reliability indicates, by definition, a greater degree of random error in the measurement (Moosbrugger, 2012), the relations at the individual level might provide a rather conservative estimation for the relation of perceived IRNO and cheating (Schmitt, 1996). In our sample, particularly the reliability of perceived IRNO was low, which might be due to its length or the breath of its content tapping into both interactions of the teacher as well as other students in the classroom. Future research should examine these relationships with a longer, more current measure of perceived IRNO as well as a more current measure of cheating behavior including new forms of cheating (e.g., using mobile phones). Another measurement-based limitation is the skewness of our cheating measures. Not surprisingly, there is a floor effect due to many students reporting not to cheat at all. This reduced variability in our variables may be another reason why correlations between perceived IRNO and cheating did not emerge as expected.

Further, we did not measure actual cheating behavior but rather relied on students’ reports. Thus, one cannot generalize the finding without considering actual cheating in class. Research indicates that there has been an increase in reported cheating in recent decades (Whitley, 1998; Jensen et al., 2002), which may be due to greater willingness to cheat or lower reluctance to report cheating. Regardless of whether students may be less reluctant to report cheating now, assessing actual cheating might still be an issue due to social desirability and fear of getting caught. However, self-reports are more advantageous regarding economical and ethical aspects: Conducting a field experiment with students and using an actual cheating measure is costlier regarding time and resources. Further, an actual cheating measure would imply a violation of personal privacy. Still, future research might focus on students’ actual cheating rather than students’ reports in order to avoid biases.Another limitation regarding the issue of biased self-reports stems from our general procedure: Even though students gave their answers to their teachers in a sealed envelope, we cannot assure that students did not answer questions about cheating in a biased manner. One can argue that students did not report their “true” cheating behavior accurately because of presumed negative consequences once their teacher knew about their cheating behavior. In our sample around 83% of participants reported to have cheated at least once, which is in line with previous research (e.g., Whitley, 1998). Thus, one can assume that students answered not more or less biased than in other research studies using self-reports. However, to reduce biased answering behavior even more, it is advisable that students hand over their answers to neutral data collectors.

Lastly, our data was cross-sectional, and we can therefore not determine causality. In general, assuming mono-causality might not be appropriate. Even though it is not assumed based on the theoretical reasoning of the present manuscript, it may be that students in a classroom where cheating is more common perceive their teacher’s IRNO to be lower. If students “get away” with cheating without the teacher noticing that their knowledge is actually stagnating or declining, students may consider the teacher less able to evaluate them based on their personal development over time. However, most of the past research on cheating in educational settings applied cross-sectional designs (e.g., Anderman et al., 1998; Tas and Tekkaya, 2010), where testing for (mono-)causality is not possible. Therefore, future research should include either a longitudinal perspective on the relationship between students’ perception of their teacher’s IRNO and their cheating behavior – to test for mono- as well as bi-causality using cross-lagged panel models – or apply experimental designs.

## Conclusion

In this study, we examined the relationship between students’ reported cheating behavior and their personal and collective perceptions of their teachers’ IRNO. We expected students to report less cheating when they perceived their teacher as having a higher IRNO. The results partially confirmed our hypothesis on the classroom level as perceived IRNO predicted the level of reported cheating behavior on written exams and on homework, but not in oral exams. Students’ personal perceptions of their teacher’s IRNO did not relate to their own cheating behavior. Based on the results, our suggestions for teachers to reduce cheating behavior in written exams and homework are simple: Teachers should aim to cultivate an IRNO and try to put criterion-based grades into perspective by providing personal feedback on development. For example, when correcting homework assignments or written exams, teachers might give brief individualized feedback to each student like “this is definitely an improvement compared to your last homework assignment.” Most importantly, teachers should focus on creating a culture of individual reference norms rather than targeting specific students who potentially perceive the IRNO as low when it comes to reducing cheating in the classroom.

## Data Availability Statement

The raw data supporting the conclusions of this article will be made available by the authors, without undue reservation.

## Ethics Statement

Ethical review and approval was not required for the study on human participants in accordance with the local legislation and institutional requirements. Written informed consent to participate in this study was provided by the participants’ legal guardian/next of kin.

## Author Contributions

TM, AN, and OD developed the study concept and contributed to the study design. AN and TM analyzed and interpreted the data and prepared the draft manuscript. OD provided critical revisions. All authors contributed meaningfully to the paper, approved the final version to be published, and agree to be accountable for all aspects of the work in ensuring that questions related to the accuracy or integrity of any part of the work are appropriately investigated and resolved.

### Conflict of Interest

The authors declare that the research was conducted in the absence of any commercial or financial relationships that could be construed as a potential conflict of interest.

## Footnotes

^1^The comparative fit indices AIC and BIC indicate that the model fit when including grade level – a factor highly correlated with age – as a covariate on the class level is worse (AIC = 5089.47; adj. BIC = 5122.06) than the model fit when not including grade level. Further, grade level had no statistically significant effect on cheating on written exams (*p* = 0.82) nor on cheating on homework (*p* = 0.85), but on cheating on oral exams (*β* = -0.48, *p* = 0.04). Another model including age as a covariate also had a worse model fit (AIC = 5755.66; adj. BIC = 5788.33), and age had no statistically significant effect (*p*s > 0.05).
